# The comprehensive effects of water-nitrogen coupling regulation on energy-saving greenhouse tomato growth and water-nitrogen utilization

**DOI:** 10.3389/fpls.2025.1644877

**Published:** 2025-09-12

**Authors:** Mingyu Zhao, Dongshuang Niu, Bo Li, Chihai Pan, Mingze Yao, Lizhen Mao, Zhanyang Xu, Lei Sun, Manman Gao

**Affiliations:** ^1^ College of Water Conservancy, Shenyang Agricultural University, Shenyang, China; ^2^ Baicheng City Water Resources Survey and Design Institute, Baicheng, China; ^3^ School of Economics and Management, Shihezi University, Shihezi, China; ^4^ Department of Foreign Languages Teaching, Shenyang Agricultural University, Shenyang, China; ^5^ College of Water Resources and Civil Engineering, China Agricultural University, Beijing, China

**Keywords:** yield, EWTOPSIS model, structural equation model, greenhouse tomato, water use efficiency, partial factor productivity of nitrogen

## Abstract

Irrigation and nitrogen application in an irrational manner can reduce tomato yield and resource use efficiency. To investigate the effects of different water and nitrogen combinations on yield, dry matter, ET, water use efficiency, and partial factor productivity of nitrogen, we conducted a tomato plot experiment from August to December in 2020 and 2021.Three irrigation levels (65%~75% ${\text{θ}}_{f}$ (W_1_), 75%~85% ${\text{θ}}_{f}$ (W_2_), 85%~95% ${\text{θ}}_{f}$ (W_3_) and four nitrogen application rates 0 (N_0_), 120 kg·hm^-2^ (N_1_), 240 kg·hm^-2^ (N_2_), 360 kg·hm^-2^ (N_3_)) were set in this experiment. We conducted ANOVA and Pearson’s correlation analysis for a number of indicators measured in the experiment, and also utilized structural equation modeling and Entropy Weighted TOPSIS(EWTOPSIS) modeling for comprehensive analysis and evaluation. The results showed that irrigation and nitrogen application had significant effects(P<0.05) on tomato fruit yield, total dry matter content, ET and water and nitrogen utilization efficiency. Increasing irrigation and nitrogen application would achieve higher yield and total dry matter content, but was not conducive to water conservation and efficient use of water and fertilizer. Structural equation modeling indicated that ET, WUE, and dry matter all had a positive effect on yield, with WUE being the main controlling factor for yield and having the greatest influence, with a path coefficient of even 0.81. The results of the EWTOPSIS model for the two-year experiment indicators showed N_1_W_3_ as the treatment with the best overall benefits. Ultimately, this study found that greenhouse tomatoes could be optimized economically in Northeast China by controlling soil water content to 85% to 95% of field capacity and applying 120kg hm^-2^ of N.

## 1 Introduction

In recent years, climate change has led to frequent occurrence of abnormal weather and unpredictable rainfall, and water resources have become increasingly important (Tabari, 2020). Correspondingly, water use in agricultural production is inefficient and many water resources are not effectively utilized (Kang et al., 2017). In the high latitude regions of the world and in the northern part of China, local farmers often grow vegetables in greenhouses in order to get a supply of fresh vegetables (Du et al., 2018; Cui et al., 2020).However, most of the production of facility vegetables is characterized by high irrigation and nitrogen application (Rahil and Qanadillo, 2015; Ding et al., 2022; Wu et al., 2022a; Bello et al., 2024), and this irrational irrigation and nitrogen application leads to serious environmental pollution problems, such as ammonia volatilization, greenhouse gas emissions, nitrate leaching, and water eutrophication (Song et al., 2018; Zhang et al., 2023b), and also leads to decreased tomato yields and lower quality (Li et al., 2023b). Most of these problems are caused by poor water and fertilizer management in greenhouses, which is not conducive to water conservation and efficient use of resources. Therefore, we should carry out scientific management of water and fertilizer in greenhouses to realize the sustainable development of facility tomato.

Drip irrigation is a high-efficiency water-saving irrigation method widely used in greenhouse tomato production (Wu et al., 2022b; Zhang et al., 2023a). Numerous scholars have proved that using drip irrigation to grow tomatoes can achieve high yields and efficient water utilization (Sun et al., 2023; Bello et al., 2024; Zhang et al., 2024). Drip irrigation combined with plastic mulch can locate water and fertilizer at crop roots through sprinkler heads, which improves the reduction of surface runoff and evaporation of plants (Zhang et al., 2023a), and it is a good irrigation measure. In addition to irrigation measures, the application of nitrogen fertilizer is also a common measure to achieve high yields. Previous studies have shown that increased application of nitrogen fertilizer can improve crop uptake of nitrogen, which in turn enhances yield and dry matter quality (Badr et al., 2016; Du et al., 2017). Water-nitrogen coupling is widely recognized to exist in the growth process of plants (Fan et al., 2022), but there is a lack of research on the mechanism of its effects. It has been reported that water and nitrogen deficits have an effect on crop yield, but their results vary in different experiments depending on soil texture and climatic conditions (Lu et al., 2019). Previous studies have shown that a rational mix of water and nitrogen is a key factor in improving crop yield and water and nitrogen utilization efficiency (Yu et al., 2023). Therefore, rational irrigation and nitrogen application under drip irrigation is particularly important (Gu et al., 2018; Yu et al., 2019). And still remains to be studied about the effect of water-nitrogen coupling effect on tomato growth and development and dry matter accumulation under greenhouse conditions.

In the past few years, scholars from various countries have been competing for the optimal water and nitrogen combinations in agricultural production. Some of the methods used are Principal Component Analysis (PCA) (Jiang et al., 2024), Gray Relevance Approach (GRA) (Wang et al., 2015), Technique for Order Preference by Similarity to Ideal Solution (TOPSIS) (Luo and Li, 2018), etc. PCA will discard part of the data during dimensionality reduction (Jiang et al., 2024), GRA mainly focuses on the geometric similarity between data sequences, with less consideration of the distributional characteristics of the data, and lacks a clear concept of the optimal solution (Tao et al., 2024), and different methods yield different results (Li et al., 2021).This requires us to use a more integrated model for the analysis. In this process, some scholars have proposed the Combined Evaluation Method (CEM), which improves the weighting of indicators on the basis of the original evaluation model (Li et al., 2021). Many scholars have thus created more comprehensive evaluation methods such as Multi-Weight Combination Evaluation Method (Yu et al., 2024), CRITIC-TOPSIS (Zhang et al., 2025b), Subjective and objective empowerment combined with the TOPSIS method, and so on. From this we can see that TOPSIS is recognized by the majority of scholars, this is because TOPSIS is not affected by the distribution of data and the sample size, the number of indicators, and can better analyze the strength of the combined impact of multiple indicators (Yu et al., 2023). Therefore, TOPSIS was selected as the main evaluation model in this study. In addition, in order to exclude the influence of subjective factors in weight allocation, we introduced the entropy weight method on this basis to obtain more reasonable weights, thereby improving the reliability of the evaluation results (Zhang et al., 2022).

Structural Equation Modeling (SEM) is also a good method to analyze the relationship between multivariables, which can not only quantitatively study the interaction between multivariables, but also fit and judge the structural model formed by multivariables together. In recent years, many scholars have used SEM for correlation analysis. Among them, (Tian et al., 2025) found that water-fertilizer coupling was found to increase Pn, Tr, and Gs of crops by increasing LAI and SPAD, which ultimately contributed to the increase of crop yields through SEM’s pass-through analysis; (Ma et al., 2025)found wheat grain yield directly influenced by irrigation and wheat variety through SEM; (Ma et al., 2024b) used SEM to elucidate the direct effect of photosynthetic parameters (Pn and Chl) and biomass characteristics (total and root biomass) on yield. It can be seen that SEM does have a wide range of applications, so SEM was used in this study to analyze a number of indicators measured in greenhouse tomatoes.

Above all, the purpose of this study is: (1) Quantitative measurements of greenhouse tomato growth indexes to investigate the effects of different water-nitrogen couplings on greenhouse tomato growth and development and water and nitrogen utilization. (2) Structural equation modeling was used for path analysis and factor analysis of the multiple indicators measured to derive the effect of multiple factors on tomato yield. (3) Based on the indicators measured by different water-nitrogen coupling treatments using the entropy weight method and TOPSIS model, a comprehensive evaluation system was constructed to derive a suitable irrigation nitrogen application strategy for the region.

## 2 Materials and methods

### 2.1 Experimental materials

**Figure 1:**
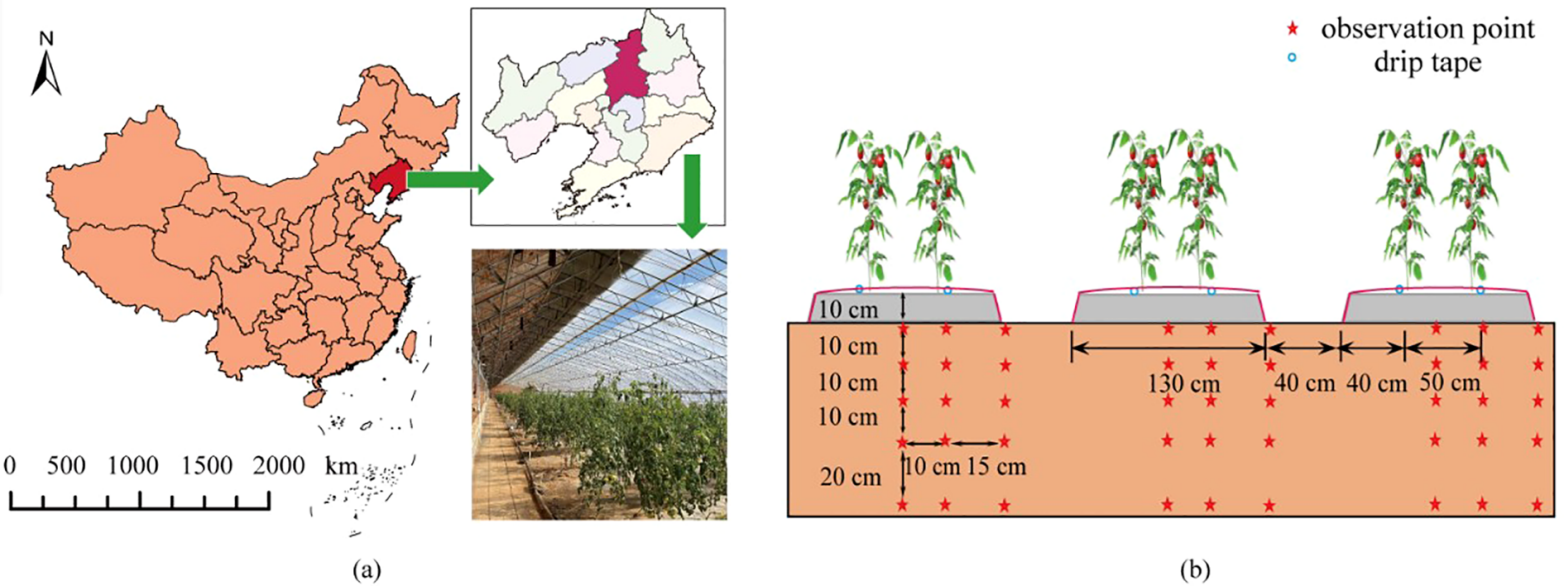
Geographical location of the test area **(a)** and moisture probe location **(b)**. The scale bar in the lower left corner of the figure represents the drawing scale of the map of China. The vertical 10cm on the right represents the vertical spacing between observation points. The horizontal values from left to right represent the horizontal spacing between observation points, the width of the plots, the distance between plots, the distance from the edge of the plots to the tomato plants, and the spacing between tomato plants, respectively.

**Figure 2:**
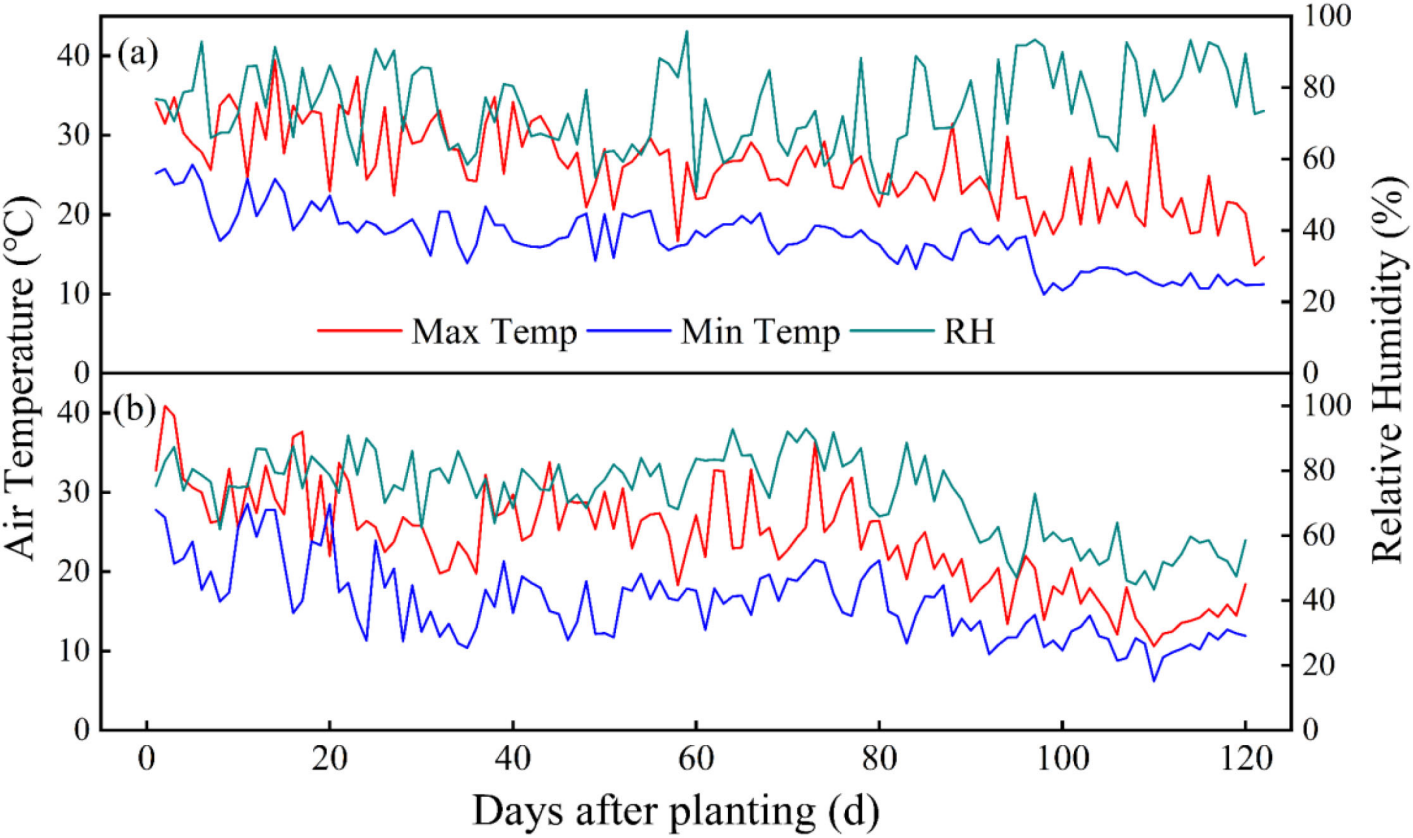
Air temperature and relative humidity in the solar greenhouse in 2020 **(a)** and 2021 **(b)**.

This study was carried out in No.43 greenhouse (41°49′N,123°34´E, 81m altitude) in the research and experimental base of Shenyang Agricultural University, which is located in the northern cold area from August to December, in 2020 and 2021, as shown in **Figure 1**, where a is the location map of the experimental area and b is the layout map of the moisture probe. The greenhouse belongs to Liaoshen III solar greenhouse, with black plastic film as the shed film and thermal insulation quilt as the thermal insulation measure. The average temperature in the greenhouse in autumn and winter is 19.46°C, the highest temperature is 39.48°C, the lowest temperature is 6.20°C, the daily average relative humidity in the greenhouse is 47.8%, and the night average relative humidity is 74.9%. The day-to-day variation of greenhouse meteorological factors during the whole reproductive period is shown in **Figure 2**. The soil in the greenhouse is brown soil, the measured physical and chemical properties of soil are shown in **Table 1**.

**Table 1:** Physical and chemical properties of the soil at the experiment site.

Soil depth (cm)	Bulk density (g·cm^-3^)	Saturated hydraulic conductivity(cm^3^·cm^-3^)	Field capacity (cm^3^·cm^-3^)	Permanent wilting point (cm^3^·cm^-3^)	pH	Total N (g·kg^-1^)	Total P (g·kg^-1^)	Total K (g·kg^-1^)	Total organic matter (g·kg^-1^)
0—10	1.28	0.35	0.28	0.09	7.93	0.42	1.07	15.70	8.20
10—20	1.30	0.34	0.29	0.10	8.18	0.43	1.22	13.71	10.05
20—30	1.31	0.34	0.27	0.09	8.16	0.44	1.31	15.08	6.32
30—40	1.32	0.35	0.28	0.09	8.17	0.29	1.35	8.19	2.13
40—50	1.31	0.35	0.28	0.09	8.15	0.43	1.21	11.85	0.59
50—60	1.32	0.35	0.29	0.09	8.15	0.43	1.15	10.20	0.46

In this experiment, tomato was used as the experimental material, and the variety was Fenguan No.1. Topping should be carried out after leaving three ears of fruit, and the daily management of greenhouse should be carried out according to local routine. The growth stage of tomato was divided into three stages: seedling stage, Flowering and fruiting stage, maturation stage. The specific time division of the reproductive period is shown in **Table 2**. From the date of planting, the growth status of tomato was observed every day during the experiment period, and the start date of each growth stage was recorded.

**Table 2:** Division of greenhouse tomato growth stage.

Growth stage		Seedling stage	Flowering and fruiting stage	Maturation stage	Total growth stage
2020	Date	8.14-9.22	9.23-11.10	11.11-12.13	8.14-12.13
	Days	40	49	33	122
2021	Date	8.11-9.10	9.11-10.30	10.31-12.08	8.11-12.08
	Days	31	50	39	120

### 2.2 Experimental design

The experimental study adopts a completely random block design, in which two factors, irrigation amount and nitrogen application amount, are determined, and three irrigation levels and four nitrogen application levels are set respectively. Each treatment was repeated three times, with a total of 36 plots, the plot area was 9.1m^2^ (7m×1.3m), the tomato planting method was double rows on large ridges, two drip irrigation belts were set up on each ridge, the flow rate of the drip head was 1.6 L·h^-1^, the spacing of the drip head was 0.4m, and the drip irrigation pressure was 0.1~0.3Mpa. A 1 m long plastic film was buried between each treatment plot to prevent lateral infiltration of soil moisture.

The upper and lower limits of irrigation are controlled for water treatment: the upper limit of water control for irrigation treatment with moderate deficit (W_1_) is 75% field capacity and the lower limit is 65%; The upper limit of water control in mild deficit irrigation treatment (W_2_) is 85% field capacity, and the lower limit is 75%. The upper limit of water control for sufficient irrigation (W_3_) is 95% field capacity, and the lower limit is 85%. All treatments were fully irrigated at the seedling stage, and deficit irrigation was started at the flowering and fruit-setting stage until the end of fruit harvesting and seedling pulling. The different water treatments were irrigated using the COMBELL moisture control system for controlled irrigation. The system consists of multiple moisture probe sensors, each probe is 30cm long and is arranged to be inserted obliquely into the two ends of the plant root system, forming an open transmission line through the two stainless steel rods (parallel rods) of the probes, and judging the water content of the soil at different depths according to the sensitivity of the dielectric constant of the medium around the stainless steel rods of the sensors. Before the start of the test, soil was collected at the corresponding depth and corrected by drying to ensure the accuracy of the data measured by the instrument. The principle is to irrigate when the soil moisture content reaches the lower irrigation limit of the treatment until it reaches the upper irrigation limit according to the reading of the moisture probe, so the irrigation time is not the same for each treatment.

Nitrogen application treatment controls the amount of nitrogen application. The amount of nitrogen applied in each treatment is as follows: N_0_:0 kg·hm^-2^; N_1_:120 kg·hm^-2^; N_2_:240kg·hm^-2^; N_3_:360 kg·hm^-2^. Before planting, soil N content was adjusted with urea as a basal fertilizer to ensure that the initial N content was the same in the area where each treatment was located. Nitrogen was applied evenly at seedling stage, early and late flowering and fruiting stage, and early maturity stage, respectively, and dissolved in water at a ratio of 4% each time, and was applied by a fertilizer pump.12 samples were treated by water and nitrogen test. The fertilizers applied were urea (total nitrogen≥46.4%) supplemented with nitrogen alone and a balanced fertilizer (20% N-P-K content), with the exact dosage calculated based on the nitrogen content of the two fertilizers and the nitrogen treatment settings. The specific timing of fertilizer application for the two-year trial is shown in **Table 3**.

**Table 3:** Experiment specific fertilizer application schedule.

Year	The first time	The second time	The third time	The last time
2020	8.24	9.29	10.15	11.14
2021	8.21	9.24	10.20	11.05

Fertilizers applied were water-soluble fertilizers, including urea, a single supplement of nitrogen, and balanced fertilizers containing 20% of nitrogen, phosphorus and potassium. Actual fertilizer application is based on actual irrigation during the year following the different treatments of crops added together with supplemental irrigation, and the timing of fertilizer application specific to each treatment may deviate by 3–5 days. The times given in the table are the dates when fertilizer was applied to most of the treatments.

### 2.3 Determination items and methods

#### 2.3.1 Determination of yield

In the mature period, the yield was picked in batches according to the processing records, and the records were kept. The multiple picking was counted according to the addition of batches, and the yield per unit area (t hm^-2^) was converted by using a 0.01kg electronic scale according to the area of each plot.

#### 2.3.2 Determination of dry matter content

Plant samples were collected in five periods, flowering and fruit setting period, first ear fruit expansion period, second ear fruit expansion period, third ear fruit expansion period and harvest period. Three plant was randomly selected for each treatment. After sampling, the stems, leaves and fruits were separately bagged and marked, and the fresh weight was weighed and recorded. After weighing, the fresh weight was put into an oven, inactivated at 105°C for 1 hour, and then dried at 75°C to a constant mass, and the dry weight was weighed and recorded.

#### 2.3.3 Determination of crop evapotranspiration

The evapotranspiration of tomatoes is calculated by water balance method, and the water balance equation is:


$${\text{ET}}_{c}=P+I+W-R-D-\Delta S$$

Formula: *ET_c_* is the stage evapotranspiration, mm; *P* is the rainfall during the period, mm; *I* is the irrigation amount during the period, mm; *W* is groundwater recharge, mm; *D* is the deep leakage, mm; Δ *S* is the change of soil moisture content, that is, the soil moisture content before the next stage of irrigation minus the soil moisture content before this irrigation. Because the rainfall in the greenhouse is neglected, the groundwater recharge is neglected, the terrain in the greenhouse is flat, a single irrigation amount cannot form surface runoff, and there is no leakage in the 60cm soil layer in the greenhouse, So final expression for crop evapotranspiration thus simplifies to:


ET=I−ΔS

Among them:


$$\text{Δ}S=1000\text{γH }(\theta_{1}-\theta_{2})$$

Formula:γis soil bulk density, g cm^-3^; *H* is the depth of soil layer, m; *θ_1_* is the initial water content (%); *θ_2_* is the water content (%) at the end of the period. Soil moisture content, soil moisture monitoring is monitored in real time by Combell moisture monitoring system, and data is recorded every 15 minutes.

#### 2.3.4 Determination of indicators

(1) Water use efficiency:


$$\text{WUE}=\frac{100\text{Y}}{\text{ET}}$$

Formula: WUE is water use efficiency, kg m^-3^; Y is the total yield, t hm^-2^; ET is the total Crop evapotranspiration, mm.

(2) Partial factor productivity of nitrogen (Zhou et al., 2023):


$$\text{PFPn}=\frac{1000\text{Y}}{\text{N}}$$

Formula: PFPn is the partial factor productivity of nitrogen, kg kg^-1^; Y is the total yield, t hm^-2^; N is the amount of nitrogen fertilizer applied, kg hm^-2^.

#### 2.3.5 Modeling of structural equations

Structural equation modeling is a multivariate statistical technique that integrates statistical methods and causality theory for analyzing complex relationships among multiple variables. It combines the advantages of path analysis and factor analysis, and is able to deal with both measurement error and the relationship between latent variables, not only to test the hypothesis of causality between variables, but also to evaluate and revise the model as a whole.

Its structure consists of two main parts, namely the measurement model: for describing the relationship between observed variables and latent variables; and the structural model: for describing the causal relationship between latent variables. The specific steps for modeling the structural equations for this test are as follows:

Step 1: Based on the relevant theories and research hypotheses, the latent variable (tomato yield) and the observed variables (other indicators) were identified and their relationships were clarified to construct an initial structural equation model.
Part 2: Determine the appropriate sample size and sampling method according to the research purpose and modeling requirements. Here we selected five indicators of tomato fruit yield, dry matter content, crop evapotranspiration, water use efficiency and nitrogen partial productivity to analyze 36 sets of data measured by 12 treatments in this experiment.
Step 3: The parameters of the model are estimated using maximum likelihood estimation (ML) to obtain the coefficients of the relationship between the observed variables and the latent variables as well as the causal coefficients between the latent variables.
Step 4: Several fit indices such as chi-square (χ^2^) to degrees of freedom (d_f_) ratio (χ^2^/d_f_<3), p-value based on the χ^2^ test (p>0.05), root mean square error of approximation (RMSEA<0.08), absolute fit index (GFI>0.9), canonical fit index (NFI>0.9), and non-canonical fit index (NNFI>0.9) were used to assess the fit of the model to the data. The definition and calculation of the indicators used therein refer to such articles (Grace, 2006; Hooper et al., 2007). Finally, based on the results of the optimal SEM model, the direct, indirect and total effects of the independent variables on the dependent variable are obtained.

The measurement models (X and Y) and structural model (η) that we used for this analysis are calculated as follows:


$$X=\Lambda_{x}\xi +\delta$$

$$\text{Y}=\Lambda_{y}\eta +\epsilon$$

η=βη+Γξ+ς

Formula: *X* is a measurable variable of *ξ*; *Y* is a measurable variable of *η*; Λ*_x_* is the factor loading matrix of x on *ξ*; Λ*_y_* is the factor loading matrix of y on η; *ξ* is the exogenous latent variable; *η* is the endogenous latent variable;*δ*, *ϵ* are the measurement errors of each measurement variable;*β* is a matrix of coefficients, meaning the direct interaction of endogenous latent variables; **Γ** is the effect of exogenous variables on endogenous variables; *ς* is the residual term.

#### 2.3.6 EWTOPSIS model

The Entropy-Weighted TOPSIS(EWTOPSIS) model is a comprehensive evaluation method combining entropy weighting method and TOPSIS model. The entropy weighting method allocates weights to each indicator, and then uses the TOPSIS method to evaluate each scheme. The advantage of the EWTOPSIS model is that it can consider multiple indicators simultaneously, avoid the limitations of single-indicator decision-making, and fully utilize the information between indicators to improve the accuracy and reliability of the evaluation. The EWTOPSIS model is a common comprehensive evaluation model, which has been used by scholars in various countries in recent years to solve the optimization problem of water and fertilizer in agriculture in their countries, as well as proved to be a reliable evaluation method (Yu et al., 2023; Zhang et al., 2023c). The specific steps for its calculation are as follows:

First is the weight calculation of the entropy weight method, which is divided into the following three main steps:

Suppose that given n treatments and m indices: $\left\{X_{1},X_{2},X_{3},\cdots X_{m}\right\}$ , the original matrix is formed as follows, where $x_{ij}$ is the value of the i th for the j index:


$$X=[\begin{array}{cccc} x_{12} & x_{21} & \cdots & x_{1m}\\ x_{21} & x_{22} & \cdots & x_{2m}\\ \vdots & \vdots & \ddots & \vdots \\ x_{n1} & x_{n2} & \cdots & x_{nm} \end{array}]$$

Step 1: Calculated index gravity p Calculate the proportion of the I-th processing of an indicator $X_{j}$ in the index, $p_{ij}$ is the proportion of the I-th processing in the j index:


$$p_{ij}=\frac{x_{ij}}{\sum_{i}^{n}x_{ij}},i=1,2,\ldots ,n;j=1,2,\ldots m$$

Step 2: Calculate information entropy E Calculate the entropy of an index $\;X_{J}$ :


$$E_{j}=-\frac{1}{\ln(n)}\sum_{i=1}^{n}p_{ij}\text{ln}(p_{ij})$$

Where n is the number of processes, $0\leq E_{j}\leq 1$

Step 3: Determine the weights of each indicator w

According to the calculation formula of information entropy, the information entropy of each index is calculated as $E_{1},E_{2},\ldots ,E_{m}$ , and then calculate the weight of each indicator through information entropy:


$$W_{j}=\frac{1-E_{j}}{m-\sum E_{j}}(\text{j}=1,2,\ldots ,\text{m})$$

The next step is the calculation of the TOPSIS model score, which is divided into the following six steps:

In order to eliminate the influence of different data index dimensions, the original matrix is standardized, and the calculation formula is as follows:


$$Z_{IJ}=\frac{x_{ij}}{\sqrt{\sum_{i=1}^{n}x_{ij}^{2}}}$$

Normalized matrix Z= $\left[\begin{array}{cccc} Z_{11} & Z_{12} & \cdots & Z_{1m}\\ Z_{21} & Z_{22} & \cdots & Z_{2m}\\ \vdots & \vdots & \ddots & \vdots \\ Z_{n1} & Z_{n2} & \cdots & Z_{nm} \end{array}\right]$

Step 2: Construct weighting matrix

In order to eliminate the subjectivity of TOPSIS method, entropy weight method is introduced to calculate the weight and improve the accuracy and objectivity of evaluation. The weighting matrix is calculated as follows:


$$Z_{ij}^{*}=Z_{ij}\cdot W_{j}$$

Finally, the weighted matrix is obtained $Z^{*}=[\begin{array}{cccc} Z_{11}\cdot W_{1} & Z_{12}\cdot W_{2} & \cdots & Z_{1p}\cdot W_{p}\\ Z_{21}\cdot W_{1} & Z_{22}\cdot W_{2} & \cdots & Z_{2p}\cdot W_{p}\\ \vdots & \vdots & \ddots & \vdots \\ Z_{n1}\cdot W_{1} & Z_{n2}\cdot W_{2} & \cdots & Z_{np}\cdot W_{p} \end{array}]$

Step 3: Calculate the optimal and worst solution

According to the positive and normalized weighted matrix, the ideal optimal solution and the worst solution are obtained, where the largest number in each column constitutes the ideal optimal solution vector, and the smallest number in each column constitutes the ideal worst solution vector:


$$Z^{*+}=Z_{max}=[Z_{1}^{*+},Z_{2}^{*+},\ldots ,Z_{m}^{*+}]$$

Among


$$Z_{i}^{*+}=\max\{Z_{1i}^{*+},Z_{2i}^{*+},\ldots ,Z_{ni}^{*+}\}$$

$$Z^{*-}=Z_{min}=[Z_{1}^{*-},Z_{2}^{*-},\ldots ,Z_{m}^{*-}]$$

among


$$Z_{i}^{*-}=\max\{Z_{1i}^{*-},Z_{2i}^{*-},\ldots ,Z_{ni}^{*-}\}$$

Step 4: Calculate the distance between each index and the optimal and worst solution

For the i th treatment, the distance between it and the optimal solution


$$d_{i}^{+}=\sqrt{\sum_{j=1}^{m}{\left(Z_{j}^{*+}-Z_{ij}\right)}^{2}}$$

The distance from the worst solution


$$d_{i}^{-}=\sqrt{\sum_{j=1}^{m}{\left(Z_{j}^{*-}-Z_{ij}\right)}^{2}}$$

Step 5: Calculate each processing score

For the i th treatment, it has a score of


$$C_{i}=\frac{d_{i}^{-}}{d_{i}^{+}+d_{i}^{\_}}$$

Step 6: Sort according to each processing score:

According to the $C_{i}$ value size of each processing, the optimal processing is obtained.

### 2.4 Data analysis

We use Microsoft Excel 2016 for data calculation; Variance analysis and multiple comparison were conducted by two factors and Duncan method. Matlab was used to calculate the weight and TOPSIS. Amos software was used to calculate the structural equation, and SPSS 22.0 was used for principal component analysis. Origin 2022 was used for drawing.

## 3 Results

### 3.1 Effects of water and nitrogen coupling on tomato growth and development and water consumption in greenhouse

#### 3.1.1 Effects of different water and nitrogen coupling on tomato yield in greenhouse

**Figure 3:**
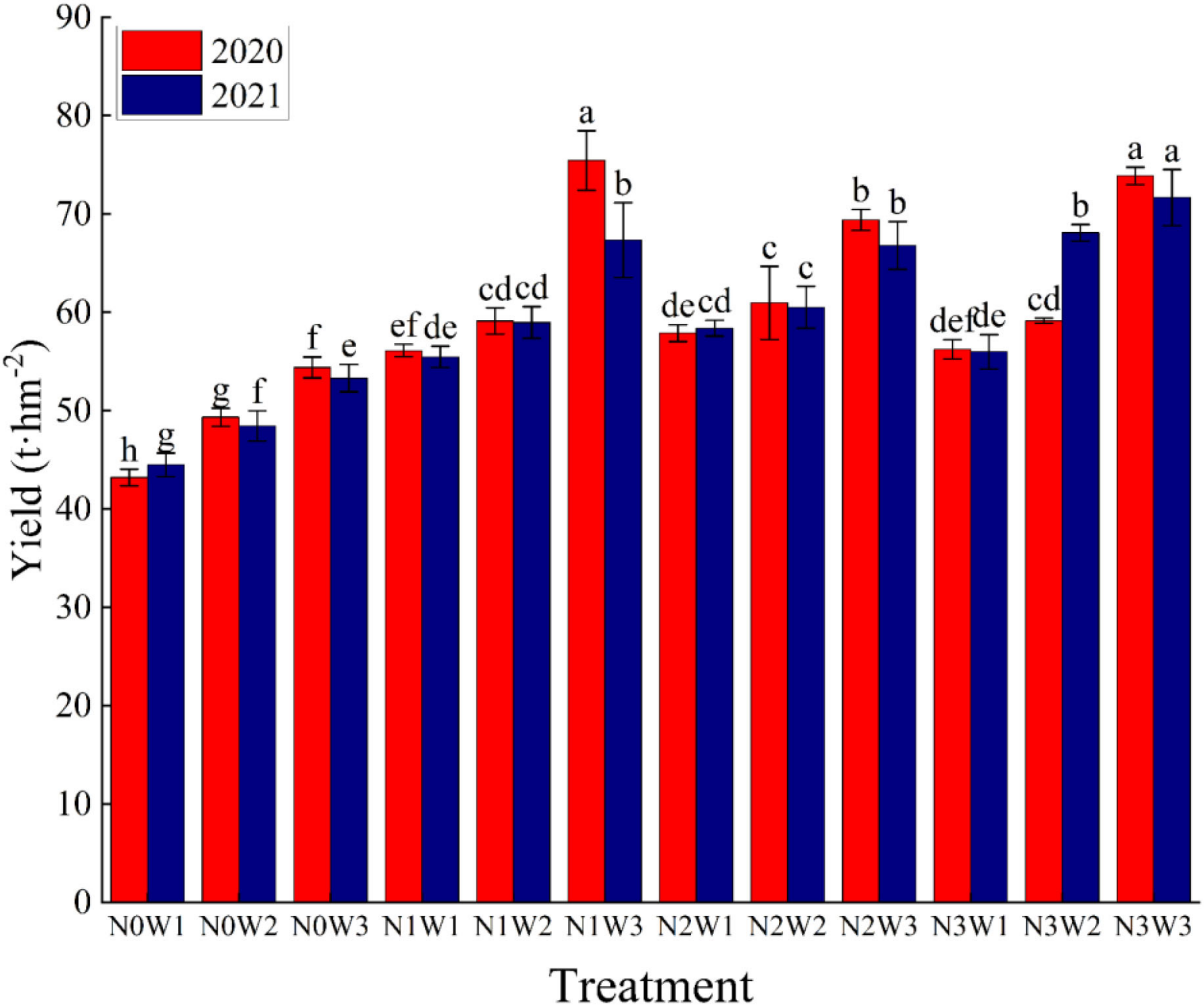
Tomato yield of different water and nitrogen combinations in 2020 and 2021. Different lowercase letters in the figure indicate their significance at the P<0.05 level, and the same letter indicates non-significance. Same as below.

The effect of different water and nitrogen combinations on tomato fruit yield in a two-year greenhouse tomato plot experiment is shown in **Figure 3**. We can see from this graph that both water and nitrogen application have an effect on yield. In addition, our analysis also revealed that the interaction effects of the single factors water and nitrogen as well as the coupled water and nitrogen on yield reached the (P<0.001) level of extreme significance in both years of the experiment (**Table 4**). In general, tomato fruit yield tended to increase with increasing irrigation, while the effect of nitrogen application on yield was found to be two fold. One is that yield enhancement is suppressed under low water and high nitrogen conditions; the other is that medium to high water conditions paired with a certain amount of nitrogen application will enhance tomato yields.

**Table 4:** Effect of different water nitrogen coupling on tomato yield, ET, WUE and PFPn in 2020 and 2021.

Treatment	2020				2021			
	Yield	ET	WUE	PFPn	Yield	ET	WUE	PFPn
N_0_	48.97b	231.01b	21.05c	—	48.74c	245.53a	19.97b	—
N_1_	63.55a	235.18b	27.02a	529.60a	60.59b	224.43c	27.05a	504.91a
N_2_	62.73a	263.36a	23.86b	261.45b	61.89b	229.60bc	27.16a	257.90b
N_3_	63.08a	256.71a	24.53b	175.22c	65.25a	240.44ab	27.25a	181.26c
W_1_	53.35c	237.48b	22.44c	288.29c	53.58c	203.77c	26.39a	282.98c
W_2_	57.14b	244.50b	23.29b	303.65b	64.58b	237.86b	25.00b	310.88b
W_3_	68.28a	257.71a	26.63a	374.33a	74.20a	263.35a	24.69b	346.21a
ANOVA								
N	***	***	***	***	***	**	***	***
W	***	***	***	***	***	***	*	***
N×W	***	***	***	***	***	***	NS	**

In the Table, Yield, ET, WUE and PFPn represent tomato fruit yield (t•hm^-2^), total evapotranspiration, water use efficiency (kg•m^-3^) and nitrogen partial factor productivity (kg• kg^-1^)in 2020 and 2021, respectively. Where different lower case letters in the same column indicate their significance at the (P<0.05) level and the same letter indicates non-significance. And *, ** and *** represent the significance of the two factors of water nitrogen on the above four indicators at the 0.05, 0.01 and 0.001 levels, NS is not significant.

The maximum values of yield in the two-year experiment were 79.43t hm^-2^ and 71.68t hm^-2^ for treatments N_1_W_3_ and N_3_W_3_, respectively. In the 2020 experiment, the average yield of N_3_ was 63.08t hm^-2^, which was 28.8% and 5.32% higher than N_0_ and N_2_, respectively, and 7.42% lower than N_1_, while the average yield of W_3_ treatment was 68.28t hm^-2^, which was 27.97% and 19.50% higher than that of W_1_ and W_2_ treatments, respectively. While in 2021, the average yield of N_3_ treatment was 65.25t hm^-2^, which was 33.88%, 7.70% and 5.42% higher than that of N_0_, N_1_ and N_2_ treatments, respectively. The average yield of W_3_ treatment was 64.78t hm^-2^, which was 20.91% and 9.79% higher than that of W_1_ and W_2_ treatments, respectively. With these data we can see that although increasing the amount of irrigation and nitrogen application gives higher yields, this does not mean that N_3_W_3_ is the optimal treatment because there may be other treatments with higher yields, such as N_1_W_3_ in 2020. Therefore, it is not the case that the highest yield can be achieved with high water and high fertilizer, but rather that we should irrigate and fertilize wisely in order to achieve higher yields while at the same time protecting the environment as well as conserving resources.

#### 3.1.2 Effects of different water and nitrogen coupling on dry matter accumulation of tomato in greenhouse

**Figure 4:**
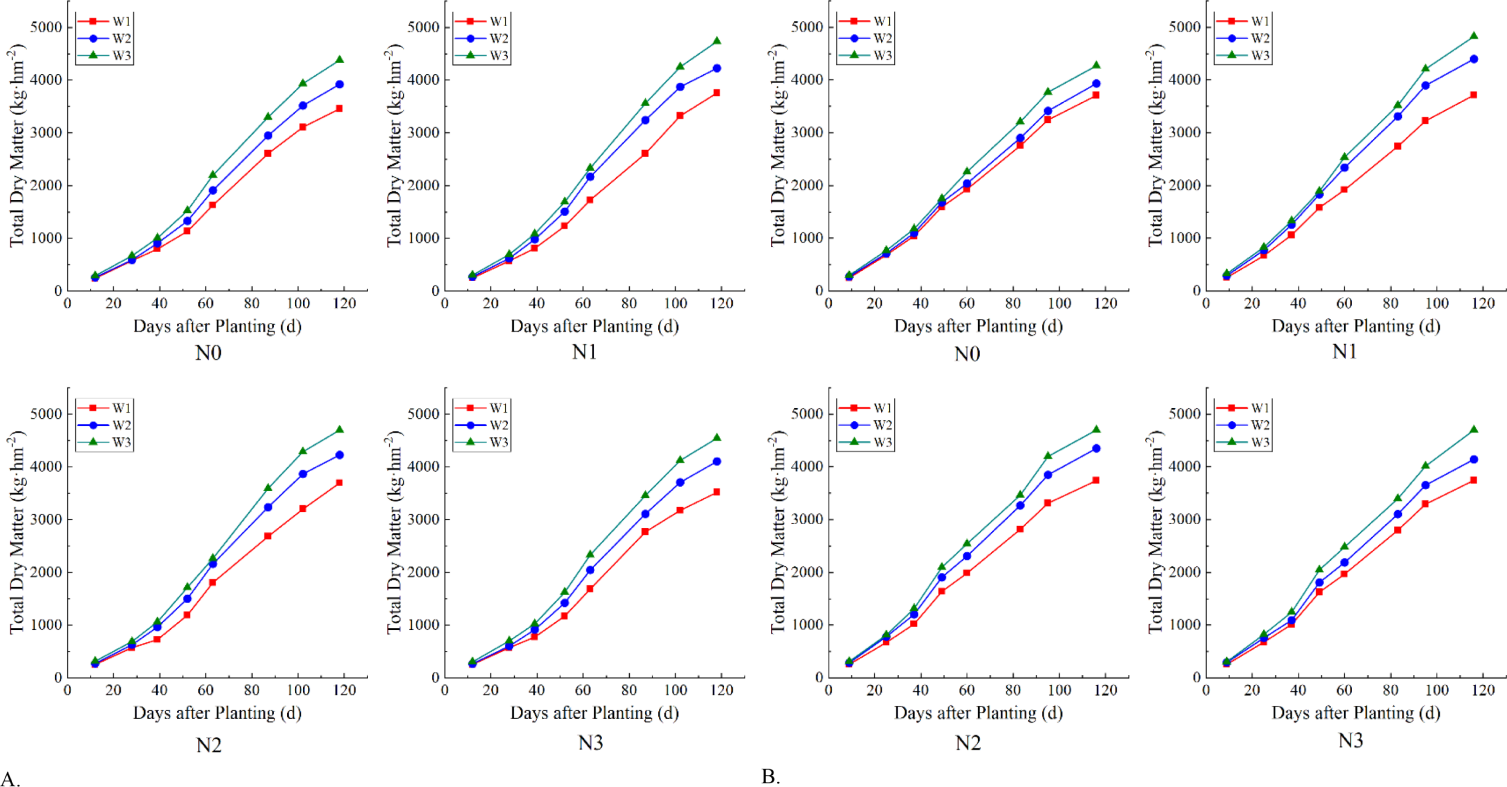
Dry matter accumulation of tomato in different water and nitrogen combinations in 2020 and 2021. **(A)** Tomato total dry matter accumulation in 2020. **(B)** Tomato total dry matter accumulation in 2021. The first five measurements of dry matter in the graph are of tomato roots, stems and leaves, and the last three times the dry matter of ripe tomato fruits was added, so the curve rose more. Different lowercase letters in the figure indicate their significance at the P<0.05 level, and the same letter indicates non-significance.

As can be seen from **Figure 4**, the accumulation of dry matter in tomato in 2020 and 2021 increased with the extension of the growing stage, and generally showed an “S” curve trend. The total dry matter measured within 80 days after tomato planting was the dry matter of tomato roots, stems and leaves, after which fruit dry matter was added for tomato maturation. The change of dry matter accumulation of tomato with the growth stage is as follows: flowering and fruiting stage > seedling stage > maturity stage. Under different nitrogen application and irrigation treatments, the dry matter accumulation of root, stem and leaf of tomato at seedling stage did not change significantly, but there was a rapid increase when entering the flowering and fruiting stage. Whereas three pickings were carried out by tomato ripening, the first one yielded more fruits and the two following ones decreased, so that the increase in dry matter of the fruits was faster and then slower.

Among all the nitrogen application treatments, the N_1_ series showed a slightly faster growth in dry matter accumulation and the maximum value that could be reached was higher, but there was no significant difference with N_2_ and N_3_, while the growth rate and limit values of N_0_ were lower than those of the other three nitrogen treatments. In 2020, the dry matter of roots, stems and leaves of the N_0_ series of treatments increased from the earliest 264.46kg hm^-2^ to 1914.17kg hm^-2^, and the total dry matter finally reached 3921.38kg hm^-2^; N_1_ increased from 277.83kg hm^-2^ to 2079.70kg hm^-2^, and finally reached 4241.10kg hm^-2^; N_2_ and N_3_ had the same pattern of change as N_1_, only decreasing in value. And the overall trend for all four in 2021 is essentially the same as in 2020. On the other hand, the dry matter accumulation pattern under different water treatment conditions was that the dry matter accumulation gradually increased with the elevation of irrigation amount, but the growth rate of all three was basically the same. Among the three sets of water treatments, the total dry matter accumulation of W_3_ was basically in the first place during tomato growth and development, except for the seedling stage when it did not differ much from the other two, and then it began to take the lead when entering the flowering and fruiting stage, and eventually reached the maximum value that was also elevated compared with the other two. Among all moisture treatments in 2021, W3 was 11.20% and 4.47% higher than W1 and W2, respectively, at the first measurement at seedling stage, and by the time of the first harvest, this difference was 22.20% and 7.85%, eventually reaching 24.04% and 9.90%.

#### 3.1.3 Effects of different water and nitrogen coupling on water consumption of greenhouse tomatoes

**Figure 5:**
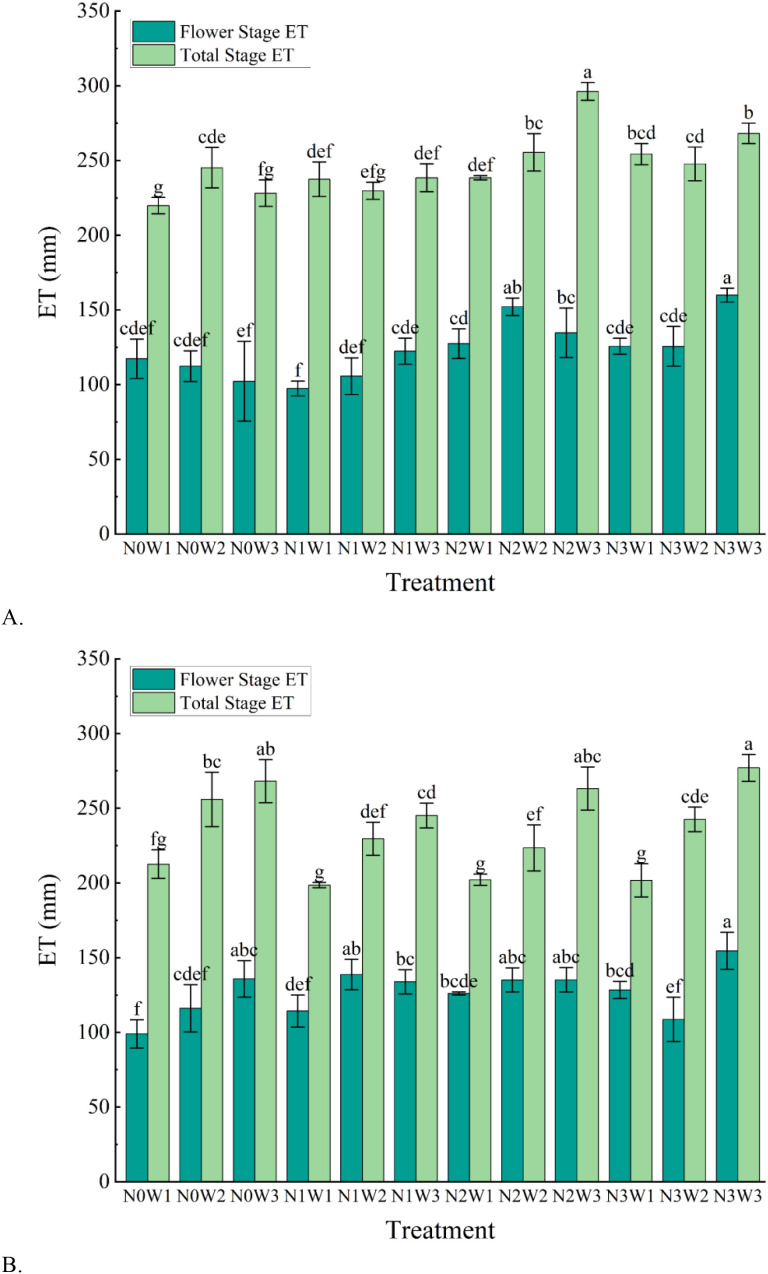
Evapotranspiration of tomato in the whole growth period with different water and nitrogen combinations in 2020 and 2021. **(A)** Evapotranspiration of tomatoes in 2020. **(B)** Evapotranspiration of tomatoes in 2021. Different lowercase letters in the figure indicate their significance at the P<0.05 level, and the same letter indicates non-significance.

The influence of evapotranspiration of tomato with different water and nitrogen treatments is shown in **Figure 5**. **Table 4** shows the effect of water and nitrogen alone and their coupling on the total evapotranspiration of tomato during the whole reproductive period, from which we can see that all three scenarios have a more significant effect on ET (P<0.01). In addition, we read the related research done by (Wu et al., 2021; Zhang et al., 2025b), and found that tomato is sensitive to water changes during flowering and fruiting (Amankwaa-Yeboah et al., 2023), so we focused on analyzing water consumption during this reproductive stage.

Overall, the results of the two-year experiment indicated that both increased irrigation and nitrogen application resulted in increased crop water consumption, but the effects were different. Increased irrigation substantially increased crop water consumption, whereas the effect of increased nitrogen application on water consumption was not as dramatic. Taking the data of 2021 as an example, the mean ET of W_1_ treatment is 203.77mm, W_2_ and W_3_ are 237.86mm and 263.35mm, and W_3_ is upgraded by 29.24% and 10.72% compared to W_1_ and W_2_. While the mean values of ET for N_0_, N_1_, N_2_ and N_3_ conditions were 224.41mm, 229.60mm, 240.44mm and 245.53mm, it can be seen that the difference between the two is not very large for N_0_ and N_1_ as well as for N_2_ and N_3_, two by two. The data for 2020 are different compared to 2021, but the overall trend is generally consistent.

Analyzing water consumption during flowering alone, we were able to find that the variation in water consumption during this reproductive period was not as pronounced as that of the full reproductive period. First of all, the water consumption of this reproductive period is generally more than 50% of the whole reproductive period, which is a good indication that the flowering period has an important influence on the growth and development of the crop, which has also been found by scholars such as (Ma et al., 2024a; Tao et al., 2024). Through the water consumption during the flowering period in **Figure 5**, we can find that the different nitrogen treatments showed an increasing and then a constant trend in water consumption. Consumption was low at N_0_ and began to increase after nitrogen was applied, but the difference was small under medium to high nitrogen application (N_2_ and N_3_) conditions. In addition, we found that a small number of treatments will decrease or even remain unchanged with the increase of irrigation, and some treatments will first increase and then decrease, but more treatments will increase the water consumption with the increase of irrigation. The three scenarios are analyzed in more detail. For example, at N_0_ in 2020, the water consumption of the three irrigation treatments was 117.35mm, 112.44mm and 102.32mm, respectively, with reductions of 4.37% and 9.89%; N_1_ are 97.39mm, 105.70mm and 122.36mm, and the W_3_ is upgraded by 25.64% and 15.77% compared to W_1_ and W_2_; The N_2_ is 127.42mm, 152.15mm and 134.73mm, which is both higher and lower; N_3_, on the other hand, is essentially the same as N_1_. And the results for 2021 are basically the same pattern of change as N_1_ in 2020 except for N_3_. Based on the above analysis of the varying degrees of increase or decrease in crop water consumption under different irrigation levels, we ultimately conclude that there is a positive correlation between water consumption during the flowering stage and irrigation levels, meaning that the higher the irrigation level, the greater the water consumption.

### 3.2 Effects of water and nitrogen coupling on water and nitrogen use efficiency of greenhouse tomatoes

#### 3.2.1 Effects of different water and nitrogen coupling on water use efficiency of tomato in greenhouse

**Figure 6:**
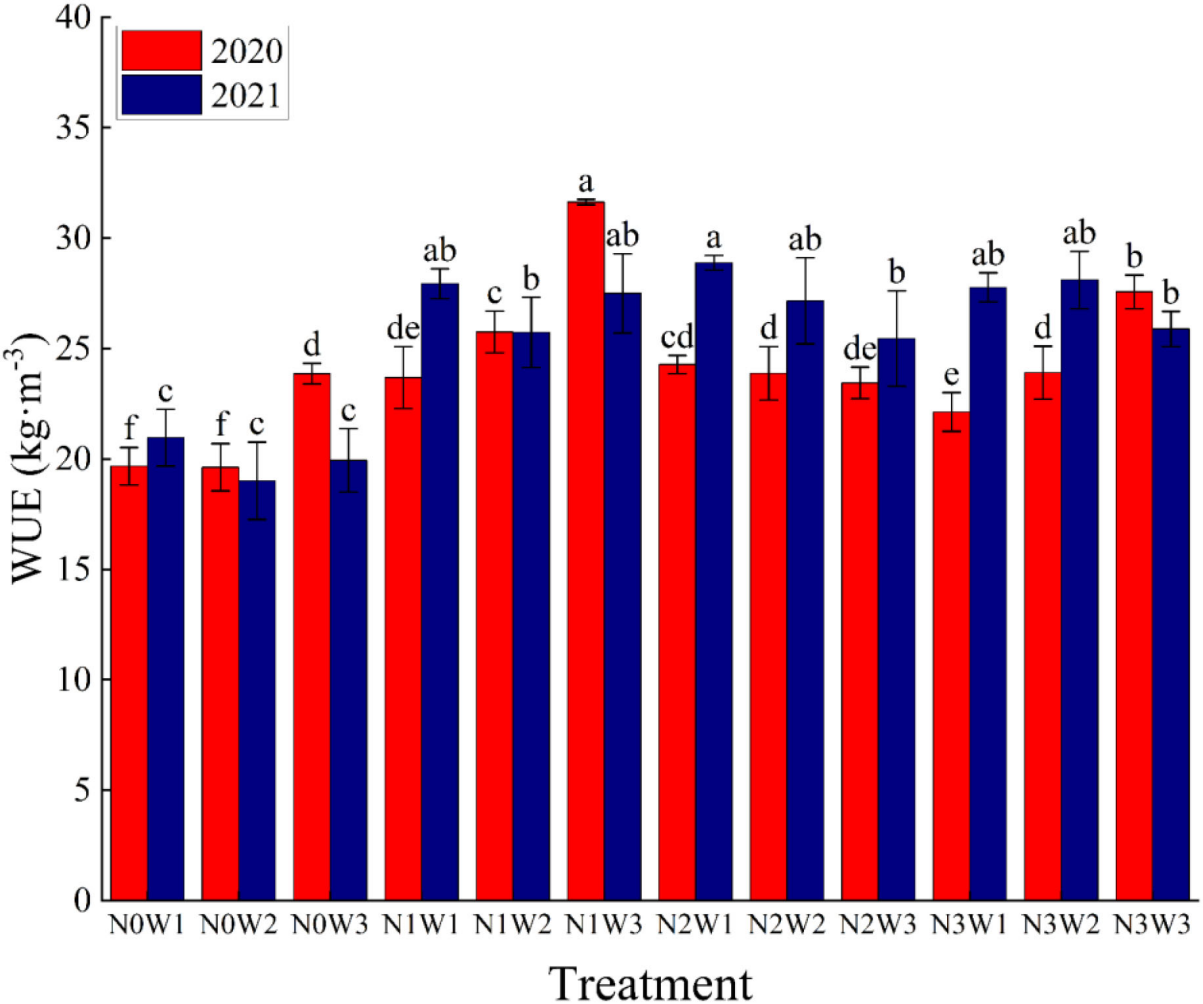
Water use efficiency of tomatoes with different water and nitrogen combinations in 2020 and 2021. Different lowercase letters in the figure indicate their significance at the P<0.05 level, and the same letter indicates non-significance.

Water use efficiency (WUE) reflects the relationship between crop yield and water consumption, and the expression of WUE is the ratio of yield to evapotranspiration. The water use efficiency of tomato in this experiment is affected by different water and nitrogen treatments, as shown in **Figure 6**. The extent to which WUE was affected by irrigation and nitrogen application and water-nitrogen coupling for the two-year experiment is shown in **Table 4**. As we can see from the table, the three have an extremely significant effect in the 2020 experiment, but the degree of effect decreases in 2021 and does not show the effect of water-nitrogen coupling on WUE.

Overall, the effect of nitrogen application on WUE showed a low level of promotion and a high level of suppression, but can be affected by differences in irrigation amount, biasing the results for some treatments. On the other hand, the effect of different irrigation amount on WUE was more complex, with both treatments that were elevated with increasing irrigation levels and treatments that were subsequently reduced. It is hypothesized that this phenomenon occurs because WUE is calculated from Yield and ET and is affected by different combinations of the two situations. However, according to previous studies, tomato WUE is supposed to decrease with increasing irrigation amount (Li et al., 2021), and this conclusion is recognized by a wide range of scholars. From **Figure 6**, we can see that the mean values of WUE in the two-year experiments were 19.62kg m^-3^~31.63 kg m^-3^ (2020) and 19.01kg m^-3^~28.89 kg m^-3^ (2021), with the minimum values being in the N_0_ treatment without nitrogen fertilizer, while the treatments where the maximum values of WUE were obtained were not fixed. However, by combining the mean values of WUE of the two-year experiments of different treatments, we can see that N_1_W_3_ has a better WUE. The results of the 2020 experiment showed that the WUE of the N_0_, N_1_ and N_3_ treatments would be enhanced with the increase in irrigation, while all the treatments in 2021 and the N_2_ treatment in 2020 had their WUE changes in the opposite direction, the latter being in line with other scholars’ studies. We therefore thought that there may have been some problems with the 2020 experiment, and after questioning and recalling it was eventually determined that the reason for this phenomenon was that there was a period of time in October when there was no effective water control due to the closure brought on by the New Crown Epidemic.

#### 3.2.2 Effects of different water and nitrogen coupling on partial factor productivity of nitrogen in greenhouse tomatoes

**Figure 7:**
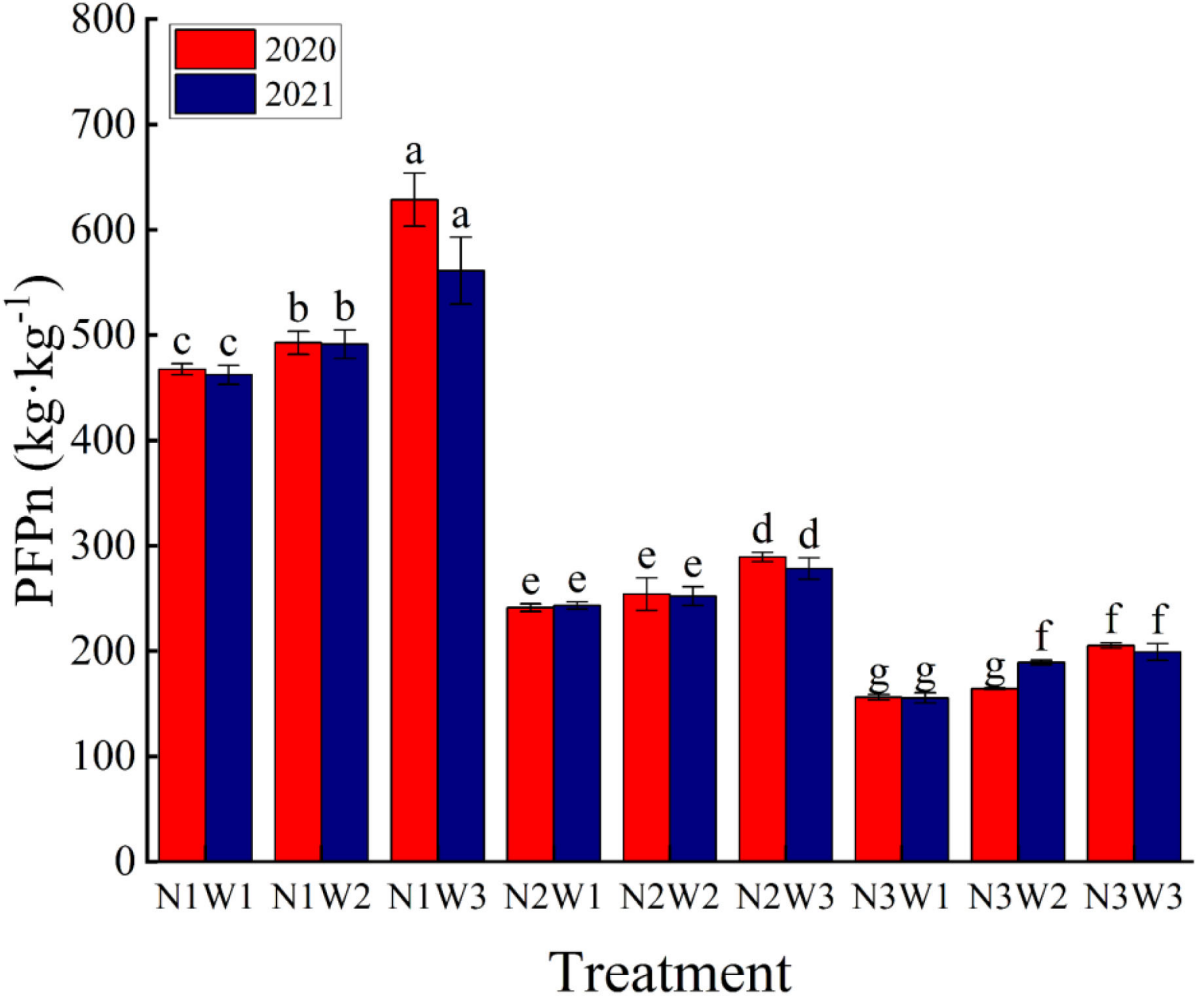
Effect of different water and nitrogen combination on partial factor productivity of nitrogen in tomato in 2020 and 2021. Different lowercase letters in the figure indicate their significance at the P<0.05 level, and the same letter indicates non-significance.

Partial factor productivity of nitrogen (PFPn) reflects the response relationship between local soil basic nutrients and nitrogen fertilizer application rate. PFPn is affected by the regulation and management of water and nitrogen. The effects of different water-nitrogen couplings on PFPn reached highly significant levels in both 2020 and 2021 (**Table 4**). As shown in **Figure 7**, increased nitrogen application significantly decreased PFPn, while increased irrigation resulted in increased PFPn.

The results of the 2020 and 2021 experiments showed that different N fertilizer application rates resulted in significant differences in crop PFPn, which was classified into three classes of 150kg kg^-1^~200 kg kg^-1^, 240kg kg^-1^~300 kg kg^-1^ and 450kg kg^-1^~630 kg kg^-1^ under N_1_, N_2_ and N_3_ conditions, respectively. Among them, the reduction of PFPn by increasing nitrogen fertilizer was the smallest 29.71% and the highest 50.63%. This indicates that a single increase in N application rate is not conducive to the effective utilization. On the other hand, increased irrigation resulted in higher crop efficiency for N fertilizer use, with PFPn enhancement ranging from 5.27% to 34.45% for W_3_ treatment compared to W_2_ and W_1_ in the 2020 and 2021 experiments, with a higher enhancement in year 20 and a decrease in year 21. These data suggest that we should apply nitrogen fertilizer judiciously to enhance crop yield and PFPn, and also by increasing irrigation to enhance crop PFPn.

### 3.3 Coupling effect of water and nitrogen on tomato yield and water and nitrogen use efficiency in greenhouse

#### 3.3.1 Correlation among yield, dry matter content, ET, WUE and PFPn of greenhouse tomatoes

**Figure 8:**
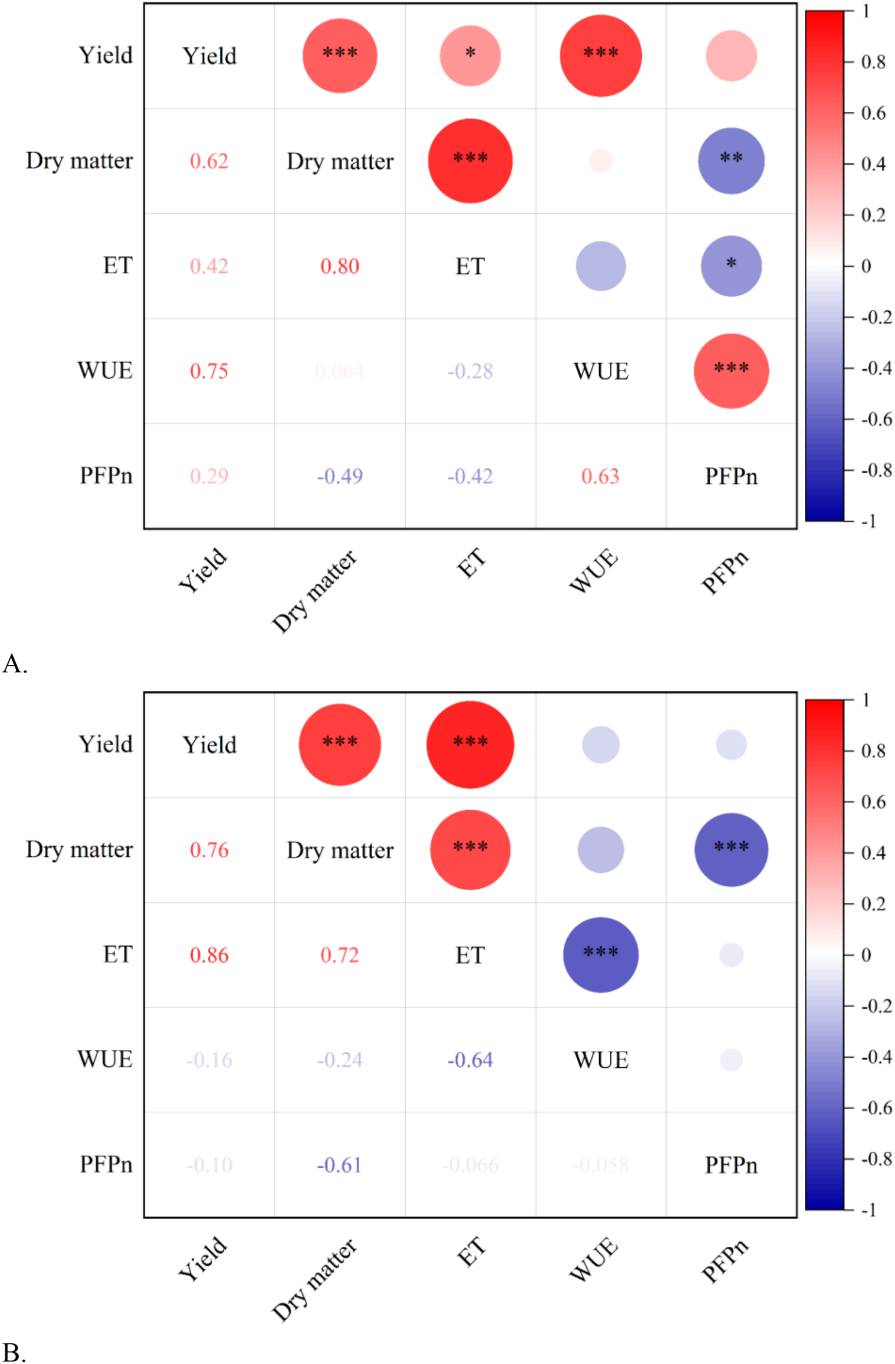
Correlation among Yield, Dry matter, ET, WUE and PFPn in 2020 and 2021. **(A)** Correlation among Yield, Dry matter, ET, WUE and PFPn in 2020. **(B)** Correlation among Yield, Dry matter, ET, WUE and PFPn in 2021. The size of the circle in the upper right corner represents the strength of the association (positive correlation represented by red color, negative correlation represented by blue color), and the number in the lower left corner represents the Pearson correlation coefficient. *, * * and * * * indicate significance at the 0.05, 0.01 and 0.001 probability levels, respectively.

Pearson correlation analysis was used to study the correlation among tomato yield, dry matter content, ET, WUE and PFPn during 2020 and 2021. The results are shown in **Figure 8**. The results of the two-year experiment showed that the positive correlation between both tomato fruit yield and dry matter and dry matter and ET reached highly significant levels (P<0.001, R>0.62). The results of the 2020 trial showed that the positive correlations between yield and WUE, WUE and PFPn also reached highly significant levels (P<0.001, R>0.63), and dry matter and ET were also negatively correlated with PFPn to some extent. In 2021, the positive correlation between yield and ET reached an extremely significant level (P<0.001, R>0.86), as did the negative correlation between ET and WUE (P<0.001, R<-0.64).

#### 3.3.2 Analysis of multi-indicator water and nitrogen coupling effects in greenhouse tomato based on structural equation modeling

**Figure 9:**
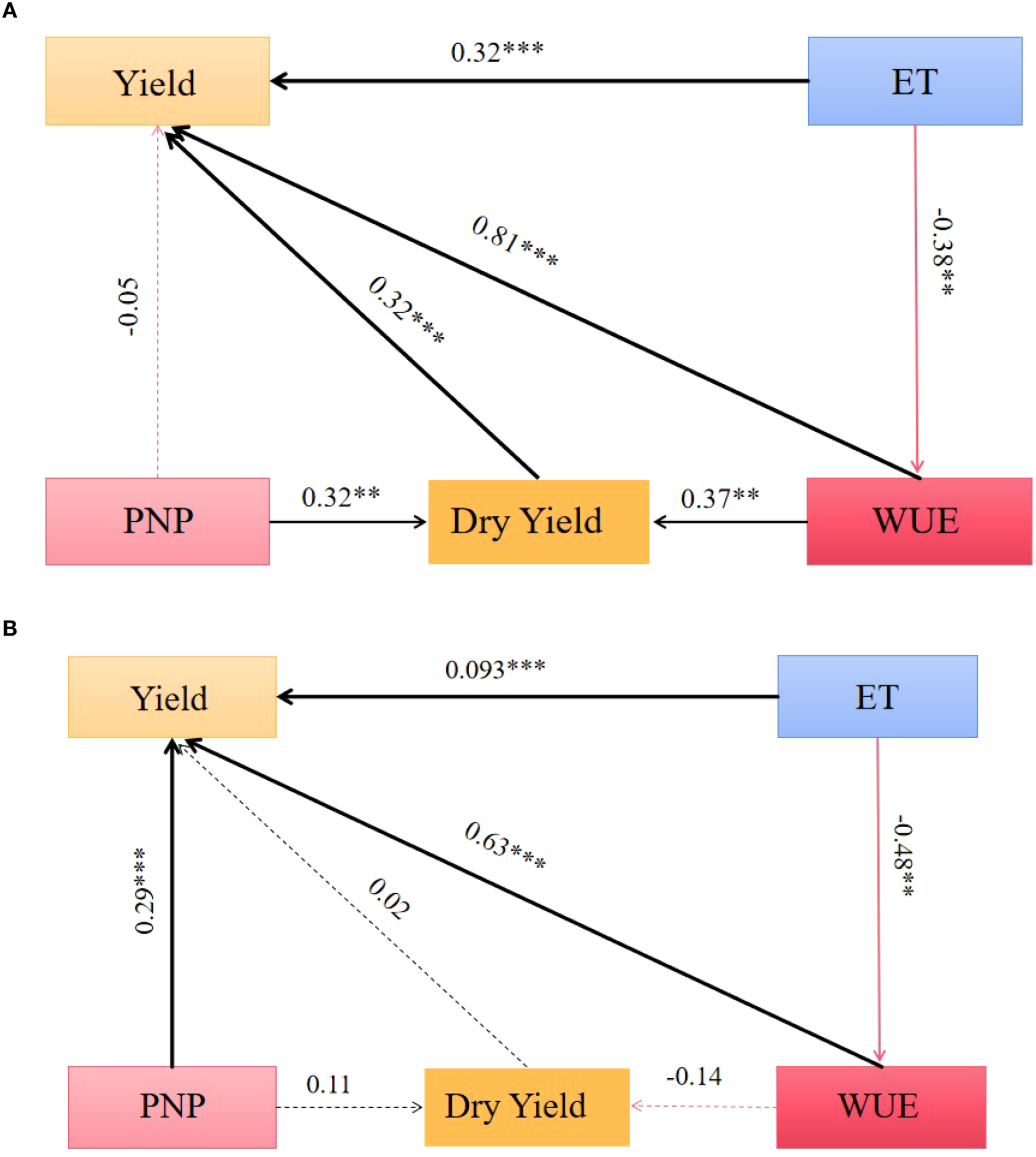
The structural equation model (SEM) shows the causal relationship among the Yield, Dry matter, ET, WUE and PFPn in 2020 **(A)** and 2021 **(B)**. The solid line in the figure indicates the presence of correlation and the dashed line indicates the absence of correlation. The width of the line segment indicates the significance of the correlation, with thicker segments indicating more significant and thinner segments indicating less significant. Black line segments indicate positive path coefficients and red line segments indicate negative path coefficients. The *, * *, and * * * above the line segments indicate significant differences at the 0.05, 0.01, and 0.001 p-levels, respectively, and the numbers indicate path coefficient.

The path relationships between tomato fruit yield, dry matter mass, evapotranspiration, WUE and PFPn were derived from the indicators measured in the two-year tomato plot experiments by path analysis and factor analysis, and the results of the calculations are shown in **Figure 9**. **Figure 9** shows the causal relationship among yield, dry matter quality, ET, WUE and PFPn in 2020 and 2021. The arrows of dry matter, ET, WUE and PFPn in 2020 and 2021 all point to the yield, indicating that there is a causal relationship between them and the yield.

In 2020, WUE, ET and dry matter point to the yield and the path coefficient is positive, indicating that WUE, ET and dry matter have a positive impact on the yield. In addition, WUE and PFPn point to the dry matter and the path coefficient is positive, indicating that WUE and PFPn have a positive impact on the dry matter quality. Among them, the path coefficient of WUE to yield is 0.81, which has the most significant influence; The path coefficient of ET pointing to WUE and PFPn pointing to yield is negative, which shows that ET has a negative impact on WUE and PFPn has a negative impact on yield. In 2021, WUE, PFPn, ET and dry matter all pointed to yield with positive path coefficients, indicating that WUE, PFPn, ET and dry matter had positive effects on yield. Among them, the path coefficient of WUE on yield was 0.63, which was the most significant; The path coefficients for ET to WUE and WUE to dry matter are both negative, indicating that ET has a negative effect on WUE and WUE has a negative effect on dry matter. There is also PFPn pointing to dry matter with a positive path coefficient, indicating that PFPn has a positive effect on dry matter.

The results of structural equation analysis for two years show that WUE has the largest path coefficient to yield, and it has a significant correlation, so WUE has the most obvious influence on yield. However, the path coefficients of the two years are not consistent, which may be caused by climate factors or environmental factors.

#### 3.3.3 Comprehensive evaluation of greenhouse tomato under coupled water and nitrogen conditions based on EWTOPSI modeling

In this study, entropy weight method and TOPSIS modeling were used to synthesize and evaluate multiple indicators measured in greenhouse tomatoes under different water nitrogen treatments over two years. Because the indicators have different dimensions and units, they cannot be directly compared and calculated. Therefore, we used the entropy weighting method to standardize and assign objective weights to the data measured by all indicators. As shown in **Table 5**. From this table, we can see that the weights of a number of indicators measured in the two-year experiment do not differ much, and only PFPn accounts for a slightly higher proportion, which to a certain extent reflects the tendency of the evaluation results.

**Table 5:** The weight of each index calculated using entropy weight method in 2020 and 2021.

Year	Yield (t·hm^-2^)	Dry matter (kg·hm^-2^)	ET (mm)	WUE (kg·m^-3^)	PFPn (kg·kg^-1^)
2020	0.152	0.182	0.197	0.21	0.259
2021	0.158	0.2	0.211	0.132	0.298

Based on the weights determined by the entropy weighting method combined with the TOPSIS model, we proceeded with the calculations. According to the positive ideal solution and negative ideal solution, find the distance d_i_^+^ from each scheme to the positive ideal solution and the distance d_i_^–^ from the negative ideal solution, and then calculate the comprehensive evaluation index C_i_ value for each evaluation object, as shown in **Tables 6** and **7**. The greater the C_i_, the better the comprehensive evaluation results of each index.

**Table 6:** Comprehensive evaluation of indexes of different water and nitrogen treatments by TOPSIS model in 2020.

Treatment	d_i_^+^	d_i_^–^	C_i_	Rank
N_0_W_1_	0.27	0.04	0.13	12
N_0_W_2_	0.25	0.06	0.19	11
N_0_W_3_	0.21	0.13	0.38	7
N_1_W_1_	0.20	0.14	0.40	6
N_1_W_2_	0.16	0.16	0.50	4
N_1_W_3_	0.28	0.29	0.79	1
N_2_W_1_	0.25	0.06	0.20	10
N_2_W_2_	0.22	0.09	0.29	8
N_2_W_3_	0.17	0.15	0.48	5
N_3_W_1_	0.22	0.07	0.25	9
N_3_W_2_	0.15	0.13	0.57	2
N_3_W_3_	0.15	0.17	0.52	3

In the table, d_i_^+^, d_i_^-^, and C_i_ represent the optimal solution, the worst solution, and Comprehensive Score through calculation for each treatment, respectively.

**Table 7:** Comprehensive evaluation of indexes of different water and nitrogen treatments by TOPSIS model in 2021.

Treatment	d_i_^+^	d_i_^–^	C_i_	Rank
N_0_W_1_	0.28	0.04	0.11	12
N_0_W_2_	0.26	0.06	0.17	11
N_0_W_3_	0.24	0.10	0.29	9
N_1_W_1_	0.18	0.17	0.49	6
N_1_W_2_	0.13	0.19	0.59	2
N_1_W_3_	0.09	0.24	0.73	1
N_2_W_1_	0.22	0.08	0.27	10
N_2_W_2_	0.19	0.12	0.38	7
N_2_W_3_	0.15	0.19	0.55	4
N_3_W_1_	0.21	0.09	0.32	8
N_3_W_2_	0.16	0.17	0.51	5
N_3_W_3_	0.15	0.19	0.57	3

In the table, d_i_^+^, d_i_^-^, and C_i_ represent the optimal solution, the worst solution, and Comprehensive Score through calculation for each treatment, respectively.

Based on the solution of the EWTOPSIS model, it can be seen that the order of merit of different water nitrogen treatments in 2020 is as follows: N_1_W_3_>N_3_W_2_>N_3_W_3_>N_1_W_2_>N_2_W_3_>N_1_W_1_

>N_0_W_3_>N_2_W_2_>N_3_W_1_>N_2_W_1_>N_0_W_2_>N_0_W_1_. In 2021 it will be: N_1_W_3_>N_1_W_2_>N_3_W_3_>N_2_W_3_>

N_3_W_2_>N_1_W_1_>N_2_W_2_>N_3_W_1_>N_0_W_3_>N_2_W_1_>N_0_W_1_>N_0_W_2_. The best treatment in the two-year results ranking was all N_1_W_3_ and the worst treatment was N_0_W_1_. This situation suggests that greenhouse tomatoes grown in northeastern China using different combinations of upper and lower irrigation limits and different nitrogen application rates have the best overall benefits when soil water content is controlled at 85%-95% of field capacity and nitrogen is applied at 120kg hm^-2^.

## 4 Discussion

### 4.1 Effects of water and nitrogen coupling on tomato yield and dry matter

Our study showed that both irrigation and nitrogen application had significant (P<0.05) effects on tomato yield (**Table 4**; **Figure 3**), and increasing irrigation significantly enhanced yield, which is the same as the findings of (Patanè and Cosentino, 2010; Yu et al., 2023; Bello et al., 2024) et al. This may be due to water deficit which leads to crop leaf closure and reduced photosynthesis (Qiu et al., 2021), inhibiting its nutrient uptake and leading to lower yields (Wang et al., 2024), in addition to the possibility that water deficit leads to a decrease in fruit water content and hence lower yields (Du et al., 2017). In contrast, adequate irrigation does not cause these phenomena, so tomato yields are improved. On the other hand, the application of nitrogen fertilizer also increased tomato fruit yield, but this enhancement was not constant, while it decreased under low water and high nitrogen conditions. However, the enhancement of yield by the application of nitrogen fertilizer was still significant compared to the absence of nitrogen fertilizer, and this phenomenon appeared to be the same as the results of the studies conducted by (Zhou et al., 2020; Yu et al., 2023). The phenomenon that high nitrogen application reduces crop yield has also appeared in the studies of (Li et al., 2017; Yu et al., 2023), which may be due to the high ionic content of the soil caused by excessive nitrogen, resulting in the obstruction of soil nutrient absorption by the root system (Matimati et al., 2014), whereas rational irrigation and nitrogen application will alleviate this adverse effect, which is conducive to the accumulation of aboveground dry matter, and ultimately to achieve high yields (Wei et al., 2018; Wang et al., 2024). In addition, rational irrigation and nitrogen application can also accelerate the coordinated growth of the root system, enhance the photosynthetic capacity, and lay the foundation for high crop yield (Wang et al., 2024). Our study also found that the N_1_W_3_ treatment had the best combined yield among the two-year experiment, but this result was not obtained at the highest N application, which is similar to the findings of (Nangare et al., 2016; Khapte et al., 2019) and others, which suggests that a single increase in N fertilizer application is not conducive to high yields.

Our study found that the accumulation of total dry matter in tomato showed a slow and then fast trend with advancing fertility, this finding similar to that of (Ma et al., 2024a). This may be because in the early stages of tomato growth, the root system is not fully developed, the plant’s ability to absorb water and mineral elements from the soil is limited (Wang et al., 2019c), resulting in the plant’s leaves are few and small (Wu et al., 2021), cannot carry out more photosynthesis, synthesize more organic matter (Tian et al., 2025), resulting in the slow accumulation of dry matter. By mid-growth, as the tomato plant grows, the leaf area increases and the number of leaves increases, and the accompanying photosynthetic capacity is enhanced accordingly (Tikoria et al., 2023), and organic matter synthesis is accelerated ultimately leading to accelerated dry matter accumulation. In addition, our study found that nitrogen application not only accelerated dry matter accumulation but also realized a numerical increase in total dry matter content. This was also found in the study by (Wu et al., 2023; Wang et al., 2024; Zhao et al., 2024).This is because nitrogen, as an essential nutrient for plant growth, influences plant growth and physiology as well as the formation of the final yield (Yang et al., 2022). Whereas, inadequate irrigation and nitrogen fertilizer application affected crop growth and development (Agbna et al., 2017; Wang et al., 2024), resulting in slower rates of dry matter accumulation. Rational nitrogen application, on the other hand, promotes the accumulation of above-ground biomass by alleviating the adverse effects of water deficit in the crop (Zhao et al., 2021). In addition, our study also found that, increasing irrigation would also promote dry matter accumulation, it did not show significant differences between irrigation treatment (**Figure 4**). This may be due to the fact that insufficient water causes crop stomata to close and photosynthesis to decrease (Qiu et al., 2021), inhibiting plant uptake of nutrients thereby reducing biomass accumulation (Bhattacharya, 2021). It is also possible that rational irrigation and nitrogen application promoted coordinated root growth, resulting in a better crop canopy structure that was able to sustain a relatively high level of photosynthesis, which ultimately contributed to the accumulation of aboveground biomass (Luo et al., 2015; Wang et al., 2024).

### 4.2 Effects of water and nitrogen coupling on tomato ET, water and nitrogen use efficiency

Our study found that different water and nitrogen treatments had a significant effect on tomato evapotranspiration (**Table 4**), and that both increased irrigation and nitrogen application resulted in increased crop water consumption. This is similar to the findings of other scholars (Lu et al., 2019; Zhou et al., 2023; Yang et al., 2024). This is due to the fact that irrigation practices increase soil water content, which in turn affects water transportation and consumption within the crop (Zhang et al., 2018). Crops in arid environments signal to the branching portion of the crop, resulting in reduced transpiration, which in turn reduces water consumption (Wang et al., 2019a). In contrast, under conditions of favorable moisture environment, the crop grows vigorously and requires more water to maintain cell expansion and various physiological activities, so water consumption will be intensified. In addition, we found that the water consumption of some of the high water treatments did not increase with the increase in irrigation amount (**Figure 5**). This may be due to the fact that under high irrigation, soil aeration is reduced and the crop root system is inhibited from absorbing sufficient water from the soil as a result of respiration in an oxygen deficient environment (Goto et al., 1997; Hu et al., 2021).It is also possible that the crop senses through the root system that the surrounding environment has sufficient water, in order to avoid excessive transpiration, closed part of the stomata resulting in reduced water consumption (P and Foulkes, 2012; Wu et al., 2023), and it is also possible that when it encounters cloudy days under high irrigation, photosynthesis in its leaves is inhibited and the energy supply for transpiration is insufficient, which affects water uptake and transport. This favors the reduction of ineffective evaporation to promote WUE and deserves our attention.

On the other hand, increased use of nitrogen fertilizer, causes accelerated plant growth, resulting in more water being consumed and absorbed by the growing root system of the crop and a subsequent increase in water consumption (Cantero-Navarro et al., 2016; Wu et al., 2023). In addition, increased nitrogen application increases the chlorophyll content in crop leaves, enhances the efficiency of photosynthesis, and results in increased transpiration and water consumption due to increasing leaf area (Jiang et al., 2024). Our study on tomato at the flowering and fruiting stage found that water consumption during this reproductive period was greater than during the whole reproductive period. This has been found to be similar in other studies by other authors (Wu et al., 2021; Zhang et al., 2025b). We also found that the water consumption pattern during this reproductive period was positively correlated with the amount of irrigation, which increased and then stabilized with the increase of N application. This may be due to the fact that during this period the plant shifts from physiological to reproductive growth and the cells need to absorb more water for volume growth and fruit enlargement (Renaudin et al., 2017; Shameer et al., 2020; Wu et al., 2021). It is also possible that during this period, the plant leaves and root system are fully developed and need to absorb more water to supply the plant to maintain a higher level of photosynthesis. The reason for the effect of water nitrogen on water consumption in this period is similar to that of the full-birth period and will not be explained here.

The results of this study showed that WUE under N_0_ and N_2_ treatments decreased with increasing irrigation, which is the same as the findings of (Zhang et al., 2025a). This may be due to over irrigation due to high irrigation which destroys soil aeration (Liu et al., 2013; Sun et al., 2024) and makes crop anaerobic respiration toxic reducing yield. In addition, the change in WUE may also be related to crop evapotranspiration, because different irrigation levels also have a certain effect on the amount of evapotranspiration, which can be proved by the study of (Lu et al., 2019). As shown in **Figure 6**, similar conclusions were obtained in our study, suggesting that moderate water deficit to the crop can enhance WUE while reducing water consumption, perhaps because drought stress causes stomatal closure and reduced transpiration in tomato leaves, which in turn improves the efficient use of water (Li et al., 2023a). In addition, the higher WUE obtained at low irrigation may also be due to the fact that deficit irrigation enhances crop yield more than leaf transpiration, which in turn is calculated from both, so WUE is elevated. On the other hand, with the increase of nitrogen application, WUE showed a tendency of increasing and then decreasing, this phenomenon is similar to the results of (Li et al., 2021), which may be due to the high nitrogen application that caused the crop to grow too fast in the early stage, and most of the nutrients were allocated to the growing organs, while the reproductive growth was reduced (Kim et al., 2008; Wang et al., 2018). Therefore, we should control the nitrogen application within a certain range to get a better WUE.

Partial factor productivity of nitrogen (PFPn) is an evaluation index used to measure crop yield and nitrogen utilization (Rasool et al., 2020). In our study, we found that the effects of different water nitrogen treatments on it reached significant levels (**Table 4**). From **Figure 7**, we can clearly see that PFPn gradually decreased with the increase of nitrogen application, and this result is the same as the findings of (Wang et al., 2019b). This may be due to the fact that excessive nitrogen disrupts the balance between plant nutrient growth and reproductive growth, resulting in lower yields (Cheng et al., 2021). In contrast, moderate application of nitrogen increases crop PFPn and yield (Spiertz, 2010).In addition, our study also found that increased irrigation increased PFPn in tomato, which may be due to prolonged water deficit resulting in reduced water uptake by the root system, which is unable to effectively utilize the nutrients in the soil for yield enhancement (Zhang et al., 2025a).or it may be due to excessive irrigation amount that results in vegetative overgrowth, reduced fruit set, poor resistance to stresses, and susceptibility to a variety of diseases, which ultimately affects the formation of tomato yields (Yang et al., 2017; Wang et al., 2024). Correspondingly, optimized irrigation improves nitrogen uptake by the crop, which in turn leads to increased yields (Bénard et al., 2009).

### 4.3 Determining the optimal treatment for greenhouse tomatoes and the relationship between the effects of multiple tomato metrics based on modeling analysis

The correlation analysis in **Figure 8** allows us to derive the relationship between the impact of the indicators and the level of significance. The results of the two-year experiment showed that there was a positive correlation between tomato fruit yield and dry matter and ET; there was also a positive correlation between yield and WUE and PFPn; however, dry matter and ET were negatively correlated with PFPn. This is due to the fact that tomato fruits are the main component of the total dry matter and the roots and leaves account for less of the dry matter, which is the same as the findings of Sun’s study (Sun et al., 2023). In addition, fruit dry weight was affected by the period and duration of water deficit (Zhang et al., 2025b), and water deficit was closely related to crop ET. The link between WUE and ET and yield is due to the fact that both are insight indicators calculated from yield, so there is a relationship.

SEM’s pass-through analysis showed that WUE, ET and dry matter positively affected yield; whereas WUE negatively affected ET. In addition, factor analysis of SEM showed that irrigation indirectly affects WUE by directly influencing ET, and WUE and PFPn then ultimately affect tomato yield by influencing dry matter accumulation. And ET and WUE can also directly affect yield. The linkages between these indicators are well documented and have been discussed in detail in Sections 4.1 and 4.2 above, and will only be discussed here in relation to why yield is used as a primary control. Firstly, the yield is the result of crop growth to the final stage, and its composition is affected by water and soil; secondly, the amount of yield determines the economic income of farmers, and is concerned by the general people and related scholars. That is why scholars from various countries use SEM in agriculture for correlation analysis taking yield into account. Previous studies related to yield have found a positive correlation between yield per plant and dry grain weight (Tang et al., 2024), and yield was also positively correlated with seed size (Taaime et al., 2023) and photosynthetic properties of crops (Tian et al., 2025). And the present study also came to a similar conclusion that WUE is an important factor affecting yield. We can further improve crop yield by increasing WUE.

The results of the entropy weight TOPSIS model evaluation and analysis in this study indicate that N_1_W_3_ is the optimal treatment. This suggests that high yields and resource conservation in greenhouse tomatoes can be achieved in northeastern China by controlling the applying 120kg hm^-2^ of N and soil moisture content to 85 to 95% of the field capacity. However, it has also been shown that tomato yields are highest under fully irrigated and moderate fertilization conditions (Yu et al., 2023);The upper limit of irrigation was 87% of the water holding capacity of the field, and a nitrogen application rate of 240kg hm^-2^ was able to obtain better tomato yield and quality (Zhang et al., 2023c); Yield, WP, PFPn and fruit quality of greenhouse tomatoes were most balanced under N application of 150kg hm^-2^ and irrigation of 70% E_pan(_ Li et al., 2021_)_. We can see that the research conclusions of various scholars are also different, which may be due to the different regions and environments as well as experimental settings, and may also be due to the different models and evaluation methods used, but it does not affect the correctness of the conclusions. Therefore, we should conduct more experiments and adopt better methods to further optimize the optimal water and nitrogen ratios in agricultural production to further enhance the efficiency of water benefits and achieve sustainable development of resources.

## 5 Conclusions

Scientific water and nitrogen management can improve crop yields and water and nitrogen use efficiency, and realize the effective use of resources. In our study, we found that irrigation and application of nitrogen fertilizer had an effect on tomato yield, dry matter quality, WUE and PFPn (P<0.05). Moderate irrigation and application of N fertilizer resulted in a maximum yield of 79.43t hm^-2^, reduced irrigation and judicious application of N fertilizer increased WUE to 31.63kg m^-3^, and reduced N fertilizer dosage and increased irrigation enhanced crop PFPn. In addition, increased irrigation and nitrogen fertilization would enhance the dry matter quality of tomato. Pearson’s correlation analysis showed strong positive correlations directly between tomato yield and dry matter mass, dry matter and ET; WUE was also positively correlated to some extent with yield and PFPn, and dry matter and ET were negatively correlated with PFPn. SEM analysis showed that WUE, ET and dry matter positively affected yield, with WUE having the deepest effect on yield. In terms of comprehensive evaluation, we used entropy weighting method combined with TOPSIS model to overcome the influence of subjective factors, and carried out comprehensive evaluation for the five indicators reflecting tomato yield and water and nitrogen utilization, and the results showed that the N1W3 treatment had the best comprehensive benefit. Growing greenhouse tomatoes in the northern cold zone, controlling soil moisture content in the field water holding rate of 85% to 95%, and applying nitrogen at 120kg hm^-2^ can obtain the best economic benefits, which is very helpful for the field management of local greenhouse tomatoes, and is conducive to guiding the majority of farmers to reasonably cultivate tomatoes in greenhouse environments.

## Data Availability

The original contributions presented in the study are included in the article/supplementary material. Further inquiries can be directed to the corresponding author.
